# Validity and Psychometric Properties of the Internet Gaming Disorder Scale in Three Large Independent Samples of Children and Adolescents

**DOI:** 10.3390/ijerph18031095

**Published:** 2021-01-26

**Authors:** Kerstin Paschke, Peter-Michael Sack, Rainer Thomasius

**Affiliations:** German Center for Addiction Research in Childhood and Adolescence (DZSKJ), University Medical Center Hamburg-Eppendorf (UKE), Martinistrasse 52, D-20246 Hamburg, Germany; p.sack.ext@uke.de (P.-M.S.); thomasius@uke.de (R.T.)

**Keywords:** gaming disorder, assessment, Internet Gaming Disorder Scale, psychometrics, validity, adolescents

## Abstract

Background: Problematic gaming has become a major health issue in children and adolescents resulting in the need for targeted valid and reliable screening instruments. This study aimed to explore the psychometric properties and criterion validity of the widely used 9-item Internet Gaming Disorder Scale (IGDS) in young gamers. Methods: Three independent samples were drawn from socio-demographically representative cross-sectional telephone surveys collected in the years 2016 (N = 762), 2017 (N = 777), and 2018 (N = 784) and analyzed separately. Results: The IGDS revealed psychometric properties suitable for screening in large samples. Cronbach’s alpha was 0.563, 0.724, and 0.778. The unidimensionality assumption was challenged. At-risk and pathological gamers compared to normal gamers reported longer digital media use and more emotional symptoms and hyperactivity/inattention with clinical relevance to medium effect sizes. The comparison of at-risk and pathological gamers indicated a partial distinction between the two problematic gaming groups. Conclusions: The IGDS could be shown to be an overall suitable and valid tool to identify pathological gamers in childhood and adolescence according to the DSM-5 criteria on a population level. However, the polythetic structure limits comparability with the recent ICD-11 criteria. At-risk gamers appeared as a heterogeneous group warranting more research.

## 1. Introduction

The use of digital games has significantly increased during recent years and especially the last months [1]. Among smartphone apps, digital games are the most popular with increasing revenue [2,3]. Children and adolescents are particularly prone to these media since a lot of games are designed to address their special interests and support strong attachment by graphics, stories, and the application of intermittent reward system techniques [4,5]. Their frequency and daily time spent with games on electronic devices significantly increased under the first lockdown measures of the COVID-19 pandemic [6]. While the neural reward system of children and adolescents is fully developed, their cognitive control mechanisms are still immature [7]. Together with the higher exposure to digital games, this makes them especially vulnerable to develop at-risk or pathological gaming behavior [8,9]. Pathological gaming is estimated by prevalence rates between 1.2% to 5.9% in European, Asian, and Australian adolescents affecting sleep and academic achievement as well as family relationships [10].

Hence, pathological gaming has become a major concern for health care professionals during the last years. This aspect has been considered by adding the Internet gaming disorder (IGD) as a “condition warranting more clinical research and experience” in the Diagnostic and Statistical Manual of Mental Disorders 5 (DSM-5) of the American Psychiatric Association (APA) in 2013 [11]. According to a systematic review focusing on children and adolescents “multiple, often interacting personal, familial, and environmental risk factors and comorbid conditions will contribute towards developing and maintaining” an IGD [12]. Pathological gamers show an average gaming time of 30 h per week [13,14]. More precisely, 9- to 19-year-old Italian children and adolescents screened with an IGD stated 22 to 27 gaming hours weekly [15]. 12- to 18-year-old Spanish males diagnosed with an IGD reported a weekly gaming time of 48 h before treatment [16].

According to the DSM-5, an IGD is assumed if at least five out of nine defined criteria have been met in the past 12 months (Table A1 in the Appendix A). Moreover, research findings and clinical observations lead to the inclusion of the diagnoses Gaming Disorder (GD) and Hazardous Gaming (HG) in the recent 11th revision of the International Classification of Diseases (ICD-11) of the World Health Organization (WHO) [17]. Given the relevance of this relatively new disorder, research and practitioners are calling for functional assessment measures, especially for screening in children and adolescents.

Available DSM-5 based assessment scales vary regarding their completeness of contents and the target age groups [10]. Self-rating instruments that have been used in young samples include e.g., the 10-item Internet Gaming Disorder Test (IGDT-10) by Király et al. [18], the 9-item Internet Gaming Disorder Scale (IGDS) by Lemmens et al. [19], the 9-item short form of the Internet Gaming Disorder Scale (IGDS9-SF) by Pontes and Griffiths [20], and the Video-Gaming Scale for Children (VGS-C) by Donati et al. [21]. The latter is an adaption of the Video-Gaming Scale for Adolescents (VGS-A) developed by the authors to specifically address the needs of children [22]. In their large review King et al. [23] identified 32 IGD assessment tools available in English that were employed in 320 studies and differed regarding “(1) conceptual and practical considerations; (2) alignment with DSM- 5 and ICD-11 criteria; (3) type and quantity of studies and samples; and (4) psychometric properties”. No single instrument was found to be clearly superior but five scales showed the best psychometric properties. One of these was the Lemmens’ IGDS, studied in 42 refereed publications from January 2010 to September 2020 (https://pubmed.ncbi.nlm.nih.gov/). It appears to be an IGD measure that is both complete regarding the coverage of DSM-5 criteria and appropriate for various age ranges as it was validated in a sample of 13 to 40 year old participants. Overall, this makes it an authorized candidate for a validation study in a target group of children and adolescents.

The IGDS uses a binary question format (yes/no) with a possible maximum sum score of 9 for all “yes” answers and higher scores indicating more problematic gaming behavior. Lemmens et al. [19] report unidimensionality according to the fit indices of a confirmatory factor analysis (CFA) and an excellent consistency with a Cronbach’s alpha of 0.93. Results of a latent-class analysis (LCA) allowed separation of light or normal gamers (scoring ≤2), heavy or at-risk gamers (scoring 3–4), and disordered [pathological] gamers (scoring ≥5). Convergent validity is supported by small to medium correlations of the IGDS sum score with psychological constructs like aggressiveness and antisocial behavior, anger control and hyperactivity, loneliness and emotional distress, low self-esteem, life satisfaction, and prosocial behavior [19,24,25]. The time spent with games could be identified as a predictor for an IGD development one year later [25]. Moreover, a problematic social media use could be shown to be significantly associated with an IGD [26].

However, the results concerning unidimensionality and good internal consistency could not be entirely replicated. In an analysis using item response theory, the criteria ‘escape’, ‘deception’, and ‘continuation’ did not contribute to diagnostic accuracy [24]. In a recent Delphi study, 29 international experts with clinical and/or research experience on IGD rated the DSM-5 criteria ‘escape’ and ‘tolerance’ as inadequate to distinguish between problematic and normal gaming [27]. According to the results of a factorial analysis on data based on the Compulsive Internet Use Scale (CIUS) [28], the item on the criterion ‘escape’ (IGDS item 8) had no association to the other items in a student sample compared to two adult samples [29].

A further validation of these aspects in different samples from a developmental science perspective is urgently required. To date, current research on IGD assessment neglects possible specifics of different age and sex groups. It is common to include children, adolescents, and adults into large-sized psychometric studies without age- or sex-related analyses and with males being usually oversampled. Even in the aforementioned informative and comprehensive systematic review on IGD assessment tools by King et al. [23], age and sex were no evaluation criteria. Furthermore, there is sparse knowledge concerning at-risk gamers. Can they be separated from normal and pathological gamers as e.g., suggested by Milani et al. [15]? Hence, the present study aimed to investigate the psychometric properties of the IGDS in children and adolescents considering different age and sex groups. The convergent validity was explored by comparing normal with at-risk and pathological gamers in terms of time spent with digital media as well as co-occurring emotional and behavioral problems.

## 2. Methods

### 2.1. Procedure

The data was collected in three independent cross-sectional surveys technically conducted by the established German marketing and social research institute *forsa* via computer-assisted telephone interviews in sociodemographic representative samples between 2016 and 2018—for details see [30,31,32]. All study participants including the parents/caregivers of participating children gave informed consent prior to inclusion. They could withdraw from the study at any time without reason. All procedures performed in this study were in accordance with the ethical standards of the institutional and national research committee, with the 1964 Helsinki declaration as well as its later amendments or comparable ethical standards.

### 2.2. Participants

From the original three surveys, only data a) of 12- to 17-year old participants who b) completed the IGDS without omissions and c) reported playing any digital games at least once a week were included in the present study. This resulted in 8.5%, 22.4%, and 21.6% excluded cases [in 2016, 2017, 2018 respectively]. Proportions of excluded 12- to 13- years old were (except for 2016) lower than of 14- to 17-years old [10.6% vs. 4.7% in 2016, 15.1% vs. 26.2% in 2017, 17.2% vs. 23.7% in 2018], so were proportions of excluded boys compared to girls [3.3% vs. 13.8% in 2016, 8.9% vs. 31.6% in 2017, 7.4% vs. 38.2% in 2018]. The main reason for being excluded was gaming less than once a week or not at all. This concerned primarily girls. Accordingly, proportions of non-gaming girls vs. boys were 24.8% vs. 3.6% in 2016, 45.0% vs. 20.7% in 2017, and 46.4% vs. 11.1% in 2018. Since failure to meet only one of the three criteria mentioned above led to exclusion from the analysis, the cases affected can be understood as *missing at random*, and their exclusion represented only a negligible bias in the samples.

Data of the 2016, 2017, and 2018 samples were analyzed separately and not combined to take advantage of three independent replication trials. This makes it more likely to identify psychometric weaknesses that would go undetected in one joint large sample. The sample size was comparable in all surveys: 2016 (N = 762), 2017 (N = 777), and 2018 (N = 784). Age groups were based on the German social code (“Sozialgesetzbuch”) which defines children by law as younger than 14 years and adolescents as younger than 18 years.

### 2.3. Measures

Time spent with digital media was measured by asking for the “average time of playing any digital game on weekends (in minutes)” in the 2016 sample, and by asking for “the daily time of using any social media” in the 2017 sample in a multiple choice format (<60 min/60–119 min/120–179/180–239 min/>240 min). This format is viewed as more reliable by marketing research practitioners than asking for exact times in minutes. Psychopathology was screened in the 2018 sample by the established Strength and Difficulties Questionnaire (SDQ) by Goodman (https://www.sdqinfo.com/) with German norms [33]. Psychometric studies on German speaking children and adolescent samples reported mixed findings on internal scale consistencies [33,34,35,36]. These will be considered in the analyses and in the discussion section.

### 2.4. Statistical Analyses

For assessing psychometric properties, item analyses and internal consistencies were performed based on the “classical test theory”. Dimensionality of the IGDS was examined by confirmatory factor analyses (CFAs), item analyses, and principal component analyses (PCA). CFA fit indices were reported in comparison to the following recommended criteria: χ^2^/df ratio < 5, root mean square error of approximation (RMSEA) < 0.08, standardized root mean squared residual (SRMR) < 0.08, Tucker–Lewis Index (TLI) ≥ 0.95, comparative fit index (CFI) ≥ 0.95 [37]. Generalized least squares (GLS) were chosen as the parameter estimation method since no assumptions of multivariate normality among the observed variables were made. Following the “known group”-paradigm of convergent validation, diagnostic scoring “0–2 (normal)” on the IGDS were compared with those scoring “3–4 (at-risk gamers)” and “5+ (pathological gamers)” by analyses of variance in metric data (ANOVA) and by non-parametric U-tests in ordinal data. Because of sample size restrictions and resulting slanted cell frequencies in group comparisons, ANOVAs were preceded by non-parametric H tests, and significance was only assumed when both tests were significant. 

All effect sizes (ES) are reported in Cohen’s d and η^2^. Considered conventions for d were: d > 0.20 = clinically relevant (small ES); 0.50 ≤ d < 0.80 medium ES, d ≥ 0.80 large ES [38]. Considered conventions for η^2^ are: η^2^ > 0.01 = clinically relevant (small); η^2^ ≥ 0.10 medium, η^2^ ≥ 0.25 large. ES were computed via Psychometrica software (https://www.psychometrica.de/) and CFAs via JASP 0.14.1.0 which is based on procedures written in the statistical programming language R (jasp-stats.org). All other computations were realized by IBM SPSS Statistics version 22 (https://www.ibm.com/de-de/products/spss-statistics).

## 3. Results

### 3.1. Sample Description

Table 1 reports the sociodemographic data of the three samples, divided by age groups and sex. About 95% of the sample attended school at the time of the interview. All samples of frequent gamers (at least once a week) consisted of a higher proportion of boys (64%) compared to girls (36%).

The distribution of IGDS scores is given in Table 2 together with the assessment outcomes. In the sample of 2016 pathological vs. at-risk gaming prevalence was 12 vs. 19%, 2017 it was 4 vs. 12%, and 2018 it was 3 vs. 11%. Prevalence of at-risk and pathological gaming was higher in boys than in girls. The total Cronbach’s alpha coefficients were 0.784 in 2016, 0.724 in 2017, and 0.514 in 2018. These are shown in Table 2 together with group-specific values. Over all samples, girls showed a mean alpha of 0.682, boys of 0.638, children (12–13 years) of 0.652 and adolescents (12–17 years) of 0.668.

Table A2 in the Appendix A shows the distribution of validation measures for time spent with digital media across the three samples (with means of typical weekend gaming time in minutes and medians of any daily social media use) and the mean SDQ total difficulties score. Moreover, the distributions of the SDQ subscale scores (Table A3) and alpha values (Table A4) are presented in the Appendix A. Due to non-satisfactory consistency values, results on the SDQ subscales ‘conduct problems’ and ‘peer relationship problems’ are reported but not interpreted. The same applies to the total difficulties scale as it is a linear combination of all four subscales and its good alpha values could be attributed merely to the scale length (20 items instead of 5), cp. [39].

### 3.2. Item Analysis and Dimensionality

Table 3 summarizes the results of confirmatory factor analyses (CFA) that were conducted to test the assumed IGDS unidimensionality. Moreover, classical item analyses were additionally conducted as PCAs, whereby the number of extractable components was restricted to k = 1. In all of the three samples the IGDS items did not clearly form one dimension. As depicted in Table 3, only RMSEA and χ^2^/df were clearly in favor of unidimensionality, items 4, 5, and 8 showed weak loadings indicating heterogeneity in item contents. According to Table 4, item 5 (escape, avoid thinking about disturbing things) yielded unacceptable values for item-total correlation (r_it_) and factor loadings (a) in all three samples. Item 8 (displacement, loss of interest) showed unsatisfactory r_it_ or low α- values in the 2018 sample. However, the consistency of the scale was not significantly improved by omitting these items: the highest increase would have been from α = 0.563 to α = 0.586 in the 2018 sample. Inspection, overall component loadings were similar, but item difficulties and total item correlations varied across samples, again indicating some heterogeneity within components.

### 3.3. “Known-Groups” Analyses

#### 3.3.1. Time Spent with Digital Media

Table 5 gives information about the average time of internet gaming and social media use. Differentiation between age groups was not useful, although desirable, due to the low cell frequencies in the gaming groups. On inspection, the gaming groups could be separated by the time spent with digital media: This was confirmed by significant overall test results with small ES (see Table 6). Planned comparisons (Helmert contrasts) of “normal vs. at-risk/pathological” revealed almost medium ES for internet gaming, and U tests showed small but clinically relevant ES for social media use. “At-risk vs. pathological” comparisons were non-significant and—with the exception of girls’ social media use―had small ES indicating a major overlap between these gaming groups in their temporal digital media use.

#### 3.3.2. Emotional and Behavioral Difficulties

The distributions of the SDQ subscale scores of the 2018 sample, depending on IGDS gaming groups, are presented in Table 7. Again, differentiation by age groups is not reasonable due to the small cell sizes. Gaming groups could be separated according to the severity of emotional symptoms and hyperactivity/inattention, as indicated by the group means. Overall, ANOVA results showed significantly small ES (Table 8). The comparisons of “normal vs. at-risk/pathological” revealed almost medium ES for emotional symptoms and hyperactivity/inattention. The outcome for girls’ hyperactivity/inattention was not significant between the gaming groups but had an almost medium ES. In the “at-risk vs. pathological” comparisons regarding emotional problems and hyperactivity, 3 out of 6 were significant, two of them in boys, indicating a possible distinction but also overlap between the two gaming groups.

Table A5 in the Appendix A shows the distribution of emotional and behavioral problems (assessed by SDQ) depending on the IGDS categories. Approximately one-third of at-risk boys simultaneously reported borderline or clinically severe emotional problems and hyperactivity/inattention. The same is true for two fifths of the girls classified as at-risk according to the German standards of Becker et al. [33]. The results of Table A5 in the Appendix A should be considered with the caveat of small sample sizes.

## 4. Discussion

The current study aimed to increase the knowledge on psychometric properties and screening capabilities of a suitable IGD instrument for children and adolescents. Young gamers are especially vulnerable to develop at-risk or pathological gaming patterns leading to the need for a valid and easy applicable screening tool. The IGDS by Lemmens et al. [19] is one of the few screening instruments to have been initially validated in adolescents and based on the DSM-5 criteria. It showed good psychometric properties in prior research.

### 4.1. Psychometric Properties

The presented results did not indicate scale consistencies in favor of either sex or age groups. Lemmens et al. [19] reported a large Cronbach’s alpha of 0.93 and unidimensionality of the IGDS. These values, however, were not replicated in our samples where alphas varied from 0.56 (sample 3) via 0.72 (sample 2) to 0.78 (sample 1) with the lower value being regarded as insufficient. It has to be noted that the age range of the original study was 13 to 40 years and, thus, included children, adolescents, and adults. Values comparable to the ones of our sample 1 and 2 were described by Koning et al. (0.74 and 0.77) [40], Van den Eijnden et al. (0.73 and 0.76) [41], and Peters et al. (0.74 and 0.77) [42] based on longitudinal studies with adolescents.

IGDS items 8 (displacement) und especially 5 (escape) did not add to scale consistencies and omitting these items did not improve scale consistencies. From a test theoretical point of view, Schmitt [43] argues that a low Cronbach’s alpha does not seriously attenuate validity, but can still deliver useful information. Internal consistency is necessary, but not sufficient for validity. Moreover, alpha reflects not only scale property but also sample attributes [39]. Looking at item difficulties in Table 4, most IGDS items in sample 2018 show less agreement (fewer “yes” responses) than in the other samples, the exception is item 5 (escape). It may be an attribute of the 2018 sample of answering reluctantly. In 2018 there was an increased interest and a higher concern regarding potential risks of digital gaming in German media and school-based prevention programs. This might have fostered a tendency to downplay gaming behavior in order to answer as socially desirable and to appear “normal” to the interviewer. Thus, this contextual influence may have contributed to the weaker psychometric results in the 2018 sample compared to the other two. In the same year Jeong et al. [44] published a study showing a significant discrepancy between self-measurements and clinically verified IGD diagnoses among adolescents with a false-negative rate of 44%.

The assumption of unidimensionality was further challenged by weak CFA- and PCA-loadings of items 5 (escape) and 8 (displacement). As reported in the introduction, the criterion escape was considered by experts to have little diagnostic value [26]. The literature finding of Besser et al. [29] that the ‘escape’-item made up a factor of its own when investigating adolescents—as opposed to adult samples—was supported in all three samples of the current study. The authors commented: “Considering the young age of the participants (…), the use of the internet for mood modification might be an age-sensitive effect that should be considered in further studies (p. 292)” [29]. For reasons of comparability, the original IGDS items should be retained for research purposes but we suggest to reword the item for the DSM-5 criterion escape in future IGD measures.

Interestingly, earlier work by the authors of this study provided initial evidence that to describe gaming disorder in children and adolescents according to the new ICD-11 criteria, the use of two factors is superior to the use of a single factor. Here, one factor reflects the cognitive-behavioral gaming symptoms and the other factor reflects impending or manifest consequences due to gaming behavior [45,46]. In contrast, an ICD-11 screening tool for adults covering four items found one underlying factor only [47]. Yet, a two-factorial solution is in line with the biaxial model of addiction where an addictive behavior is defined as pathological only when both specific symptoms and adverse outcomes occur [48]. The IGDS includes functional impairments by two out of the nine symptoms (according to DSM-5 criteria ‘problems’ and ‘conflict’), weakly loading on the main IGDS factor in all three samples. Since the scale evaluation follows a polythetic principle with every item being weighted equally, an IGD can be assumed without any impairment symptoms. Esposito et al. [49] argue for a careful consideration of monothetic or different polythetic questionnaire evaluations.

### 4.2. Convergent Validity

According to the “known group”-paradigm of convergent validation, the groups of normal, at-risk and pathological gamers were separable by ANOVAs, as well as by non-parametric H tests and U tests. In at-risk and pathological gamers compared to normal gamers, “typical weekend gaming time” was longer with clinically relevant effect sizes (2016 sample), and percentage of “4+ hours daily social media use” was larger (2017 sample).

Emotional and behavioral assessment via SDQ German norms [33] revealed emotional symptoms to be much more pronounced in at-risk/pathological gamers than in normal gamers (2018 sample) with mainly medium effect sizes. The computed ES correspond to correlation coefficients reported by Lemmens et al. [19] and Wartberg et al. [26] varying between r = 0.227 (d = 0.466) and r = 0.311 (d = 0.654). By structural equation modeling in a cross-lagged panel design, Wartberg et al. [26] found the following variables to predict IGD in N = 955 children aged 12–14 years after one year: male sex, IGD at initial assessment, as well as hyperactivity/inattention and self-esteem problems. Accordingly, it can be assumed that “at-risk” adolescents could develop a manifest pathological gaming disorder if clinically relevant hyperactivity/inattention and/or emotional symptoms are present, and no clinical intervention takes place.

Exploratory testing for group differences between at-risk gamers and pathological gamers yielded mixed results. This is conclusive because “at-risk” gamers form a heterogeneous group. However, an increased psychopathologic burden was found in this group which is worth further clinical evaluation. Thus, for clinical purposes, investigating frequent gamers by applying a cut-off score of 5+ only does not seem to be satisfying. Even an IGDS score of 3+ seems to indicate an elevated risk of co-occurring emotional and behavioral problems. However, it has to be kept in mind, that by reducing the IGDS cut-off, the chance of detecting at-risk gamers will be increased while the risk of misclassifying regular gamers as problematic will be elevated. Accordingly, Colder Carras and Kardefelt-Winter [48] argue for a careful consideration of both addiction-related symptoms and gaming-related impairments [48]. In their large-scale investigation of 7865 adolescent European gamers, 2.2% of the sample reported symptoms and impairments while 30.9% of the adolescents would have been misclassified based on their personal problems only. Whereas 23.6% of “concerned” gamers indicated only a few addiction symptoms but a high level of impairments, 7.3% of “engaged” gamers stated many addiction symptoms but only a few impairments. While the first group might resemble adolescents with higher emotional and behavioral problems with accompanied non-addictive gaming, the latter group might correspond most likely to the group of ICD-11 hazardous gamers. A distinction between these two groups is not possible by the IGDS.

### 4.3. Limitations

In the present study, comparisons between the gaming groups were limited due to low disorder prevalence, although gender-, sex-, and age-sensitive research on internet gaming is desirable. Future studies should address this issue by an oversampling of pathological and at-risk gamers. The present findings are based on cross-sectional survey data. Thus, no conclusions can be drawn about symptom stability and retest reliability. A further shortcoming of the current study is the missing criterion validity with objective markers such as logged usage times. Furthermore, to the best of our knowledge, the IGDS has not yet been validated against clinical evaluation in adolescents which is the gold standard when pursuing diagnostic purposes. Therefore, the IGDS is suitable for epidemiological research but not adequate for individual assessment. Finally, IGDS results cannot be easily transferred to evaluate a gaming disorder according to the ICD-11 criteria and there is no clear equivalent to ICD-11 hazardous gaming. Thus, studies comparing both diagnostic approaches would be of great interest.

## 5. Conclusions

The current study is the first to investigate the psychometric properties and the criterion validity of an established IGD screener in three large independent representative samples of children and adolescents. Since IGD in this young target group is of high clinical relevance, a suitable and easy to administer screening tool is urgently needed. Based on the present findings, the use of the IGDS by Lemmens et al. [17] in children and adolescents as a valid screening tool on a populational level could be supported. However, the assumption of unidimensionality was challenged. Normal gamers could be reliably differentiated from at-risk and pathological gamers. However, the heterogeneous group of at-risk gamers could not be further evaluated due to scale limitations. Since the IGDS is a polythetic tool with equal weight of each item, the presence of gaming-associated impairments is not necessary for IGD classification. Along with the missing equivalent of hazardous gaming criteria, a direct comparison of the gaming groups with the new ICD-11 definitions is limited.

## Figures and Tables

**Table 1:** Sociodemographic data of the three samples employed in this study.

Sample	Age	Sex	School	Job-Training	Other ^a^	Total
2016	12–13	Girls	133	---	---	133
		Boys	150	---	---	150
	14–17	Girls	198	17	7	222
		Boys	236	18	3	257
Total %			717 94.1	35 4.6	10 1.3	762 100
2017	12–13	Girls	129	---	---	129
		Boys	163	---	---	163
	14–17	Girls	175	8	5	222
		Boys	271	21	5	257
Total %			738 95.0	29 3.4	10 1.3	777 100
2018	12–13	Girls	108	---	---	108
		Boys	162	---	---	162
	14–17	Girls	162	11	4	222
		Boys	303	29	5	257
Total %			735 93.8	40 5.1	9 1.1	784 100

Notes. ^a^ Other is study, military/social service, job-seeking, or job.

**Table 2:** Internet Gaming Disorder Scale (IGDS) scores, assessments, and consistency coefficients across the three samples employed ^a^.

Sample	Age Group	Sex	^b^ Sum Score		Cronbach’s Alpha	^c^ Portion of at-Risk Gamers		^c^ Portion of Pathologic Gamers	
2016	12–13	Girls	1.56	(1.84)	0.754	7.1	(20)	3.9	(11)
		Boys	2.71	(2.17)	0.703	15.9	(45)	10.2	(29)
	14–17	Girls	1.01	(1.58)	0.784	3.8	(18)	2.5	(12)
		Boys	2.38	(2.20)	0.770	12.7	(61)	8.8	(42)
Total			1.90	(2.08)	0.778	18.9	(144)	12.3	(94)
2017	12–13	Girls	0.71	(1.34)	0.756	2.7	(8)	1.0	(3)
		Boys	1.60	(1.65)	0.613	9.6	(28)	3.1	(9)
	14–17	Girls	0.61	(1.05)	0.600	1.9	(9)	0.4	(2)
		Boys	1.60	(1.71)	0.646	9.9	(48)	4.1	(20)
Total			1.21	(1.56)	0.724	12.0	(93)	4.4	(34)
2018	12–13	Girls	0.75	(1.10)	0.514	3.3	(9)	0.4	(1)
		Boys	1.45	(1.56)	0.570	10.4	(28)	2.6	(7)
	14–17	Girls	0.74	(1.15)	0.682	2.3	(12)	0.4	(2)
		Boys	1.21	(1.37)	0.528	7.6	(39)	2.7	(14)
Total			1.09	(1.36)	0.563	11.2	(88)	3.1	(24)

Notes. ^a^ based on *N =* 762 for 2016, *N =* 777 for 2017, *N =* 784 for 2018; ^b^ group mean (standard deviation); ^c^ percentages (frequencies).

**Table 3:** Results of confirmatory factor analyses of the Internet Gaming Disorder Scale IGDS in three different samples plus fit indices.

Index	2016	2017	2018	Cut-off
χ^2^/df	82.688/27 = 3.063 *p* < 0.001	50.563/27 =1.872 *p* = 0.004	42.656/27 = 1.580 *p* = 0.028	<5
CFI	0.768	0.885	0.903	≥0.95
RMSEA	0.060	0.034	0.027	<0.08
TLI	0.768	0.847	0.871	≥0.95
**Items**	**Loadings**			
1. Preoccupation	0.233	0.229	0.194	
2. Tolerance	0.280	0.205	0.184	
3. Withdrawal	0.303	0.179	0.147	
4. Persistence	0.215	0.115	0.092	
5. Escape	0.096	0.159	0.092	
6. Problems	0.231	0.142	0.112	
7. Deception	0.184	0.111	0.102	
8. Displacement	0.172	0.099	0.035	
9. Conflict	0.148	0.055	0.071	

Notes. CFI = comparative fit index, RMSEA = root mean square error of approximation, TLI = Tucker–Lewis-index (non-normed fit index). All item loadings *p* < 0.001.

**Table 4:** Item analyses of the Internet Gaming Disorder Scale IGDS in three different samples.

Sample	2016			2017			2018		
Item	*p (i)*	*r_it_*	*a*	*p (i)*	*r_it_*	*a*	*p (i)*	*r_it_*	*a*
1. Preoccupation	19.0	0.488	0.617	19.5	0.463	0.654	13.4	0.404	0.648
2. Tolerance	27.8	0.493	0.613	16.6	0.440	0.623	12.9	0.369	0.632
3. Withdrawal	24.4	0.572	0.676	14.4	0.400	0.590	13.3	0.295	0.537
4. Persistence	18.2	0.473	0.642	14.0	0.261	0.414	12.4	0.232	0.389
5. Escape	26.9	0.202	0.255	26.4	0.290	0.451	27.8	0.152	0.283
6. Problems	24.8	0.468	0.627	12.1	0.344	0.534	13.5	0.257	0.454
7. Deception	17.5	0.417	0.528	8.8	0.319	0.494	7.5	0.277	0.511
8. Displacement	13.4	0.467	0.549	6.5	0.323	0.499	4.1	0.144	0.272
9. Conflict	9.0	0.446	0.595	2.2	0.296	0.471	4.2	0.289	0.482

Notes. Item difficulty *p (i)*; item-total correlation *r_it_* part-whole corrected; loadings *a* according to principal component analyses restricted to *k* = 1 component.

**Table 5:** Time spent with gaming (2016 sample, N *=* 762) and social media (2017 sample N *=* 777), depending on IGDS score.

Sex	IGDS Score	^a^ Typical Weekend Gaming Time (Minutes)		^b^ Daily Social Media Use 4+ Hours	
Girls	Normal	129.95	(85.78)	3.5	(23)
	At-risk	162.24	(104.38)	4.3	(4)
	Pathological	193.70	(93.12)	8.8	(3)
Boys	Normal	184.02	(103.47)	14.2	(92)
	At-risk	236.18	(125.12)	30.1	(28)
	Pathological	237.89	(130.70)	41.2	(14)
Total	Normal	153.68	(97.63)	17.7	(115)
	At-risk	216.67	(124.03)	34.4	(32)
	Pathological	227.07	(123.59)	50.0	(17)

Notes. ^a^ Group mean (standard deviation); ^b^ percentages (frequencies). Results of statistical significance tests are given in Table 6.

**Table 6:** Time spent with gaming (2016 sample) and social media (2017 sample): results of significance tests and effect sizes for Table 5.

**Typical weekend gaming time**									
Univariate analyses of variance						Helmert contrasts *p*; effect size *d*			
		*df*	*F*	*p*	*η* ^2^	Normal vs. at-risk/pathologic		At-risk vs. pathologic	
2016	Girls	2	7.21	0.001	0.039	<0.001	0.449	0.179	0.252
	Boys	2	10.67	<0.001	0.050	<0.001	0.450	0.923	0.143
	Total	2	32.77	<0.001	0.079	<0.001	0.577	0.481	0.084
**Daily social media use 4+ hours**									
^a^ Kruskal–Wallis H-tests						^b^ Mann–Whitney U-tests *p;* effect size *d*			
		*df*	*F*	*p*	*η* ^2^	Normal vs. at-risk/pathologic		At-risk vs. pathologic	
2017	Girls	2	19.63	0.001	0.489	0.002	0.212	0.274	0.536
	Boys	2	8.99	0.011	0.250	0.007	0.205	0.373	0.177
	Total	2	31.22	<0.001	0.397	<0.001	0.269	0.149	0.240

Notes. ^a^ Kruskal–Wallis H-tests are exact. ^b^ Mann–Whitney U-tests are exact.

**Table 7:** Distribution of Strength and Difficulties Questionnaire (SDQ) subscales in the 2018 sample, depending on IGDS score.

Sex	IGDS Score	^a^ Emotional Problems		^b^ Conduct Problems		Hyperactivity/Inattention		^c^ Peer Relationship Problems	
Girls	Normal	2.73	(2.18)	1.15	(1.11)	2.82	(2.12)	1.88	(1.54)
	At-risk	4.33	(3.09)	1.90	(1.70)	4.10	(1.92)	2.90	(1.61)
	Pathological	6.00	(2.65)	1.67	(2.08)	2.00	(3.46)	2.33	(2.31)
Boys	Normal	1.51	(1.48)	1.14	(1.11)	2.60	(1.95)	1.72	(1.46)
	At-risk	2.42	(2.18)	1.84	(1.47)	3.84	(2.26)	2.27	(1.18)
	Pathological	3.95	(2.13)	1.71	(1.59)	4.95	(2.64)	2.71	(2.31)
Total	Normal	1.99	(1.88)	1.14	(1.11)	2.68	(2.02)	1.78	(1.49)
	At-risk	2.88	(2.54)	1.85	(1.52)	3.90	(2.18)	2.42	(1.79)
	Pathological	4.21	(2.25)	1.71	(1.60)	4.58	(2.84)	2.67	(2.26)

Notes. ^a^ Group mean (standard deviation). Alphas ^b^ not acceptable or ^c^ not satisfactory.

**Table 8:** SDQ subscales scores (2018 sample): results of significance tests between IGDS gaming groups, Helmert contrasts and effect sizes for Table 5.

Univariate ANOVA Results					Helmert Contrasts and Effect Sizes			
					Normal vs. at-Risk/Pathological		At-Risk vs. Pathological	
	*df*	*F*	*p*	*η* ^2^	*p*	*d*	*p*	*d*
Emotional problems								
Girls	2	7.79	0.001	0.052	0.001	0.610	0.233	0.616
Boys	2	29.42	<0.001	0.106	<0.001	0.595	<0.001	0.716
Total	2	21.28	<0.001	0.052	<0.001	0.480	0.003	0.577
^a^ Conduct problems								
Girls	2	4.29	0.015	0.030	0.084	0.439	0.743	0.117
Boys	2	11.57	<0.001	0.045	<0.001	0.467	0.682	0.078
Total	2	16.08	<0.001	0.040	<0.001	0.460	0.596	0.091
Hyperactivity inattention								
Girls	2	3.80	0.023	0.026	0.733	0.466	0.110	0.633
Boys	2	22.43	<0.001	0.083	<0.001	0.654	0.028	0.438
Total	2	21.87	<0.001	0.053	<0.001	0.593	0.150	0.253
^b^ Peer relationship problems								
Girls	2	4.32	0.014	0.030	0.132	0.577	0.551	0.256
Boys	2	9.22	0.001	0.028	<0.001	0.352	0.252	0.202
Total	2	9.79	<0.001	0.024	<0.001	0.404	0.492	0.114

Notes. Alphas ^a^ not acceptable or ^b^ not satisfactory.

## Data Availability

The data presented in this study are available from the corresponding author upon reasonable request.

## Appendix A

**Table A1:** Brief overview of diagnostic criteria for the internet gaming disorder (IGD).

Label	Criterion
A.	Mental preoccupation with gaming
B.	Withdrawal symptoms
C.	Tolerance/increase of dosage (gaming time)
D.	Failures to gain gaming control
E.	Loss of previous interests or prior hobbies
F.	Continuation of gaming despite insight into adverse consequences
G.	Lying to significant others in respect of factual gaming
H.	Gaming in order to regulate negative moods (‘escape’)
I.	Elevated risk of losing an important social relationship (job/education)

Notes. For assuming an IGD, a person’s gaming behavior must have matched at least 5 out of these 9 criteria in the past 12 months. See details in APA [11].

**Table A2:** Distribution of validation measures across the three samples ^a^.

Age Group	Sex	^b^ Typical Weekend Gaming Time (Minutes)		^c^ Daily Social Media Use	^b d^ SDQ Total Difficulties Score	
		2016		2017	2018	
12–13	Girls	120.11	(59.71)	120–179 min	8.10	(4.47)
	Boys	178.40	(105.53)	120–179 min	8.14	(4.47)
14–17	Girls	147.97	(102.62)	180–239 min	9.47	(4.70)
	Boys	223.70	(120.50)	120–179 min	7.47	(4.28)
Total		174.64	(110.83)	120–179 min	8.16	(4.52)

Notes. ^a^ Sample 2016: N = 762, sample 2017: N = 777, sample 2018: N = 784; ^b^ group mean (standard deviation); ^c^ median category, maximum category would be 240+ minutes; ^d^ distribution of Strength and Difficulties Questionnaire (SDQ) subscales are given in Table 7 (main text); maximum score would be 40.

**Table A3:** Distribution of SDQ validation measure in the 2018 sample (N = 784).

Age Group	Sex	^a^ Emotional Symptoms		Conduct Problems		Hyperactivity/Inattention		Peer Relationship Problems	
12–13	Girls	2.01	(1.87)	1.30	(1.26)	3.30	(2.36)	2.24	(1.69)
	Boys	1.81	(1.71)	1.33	(1.39)	3.33	(2.28)	1.91	(1.59)
14–17	Girls	3.41	(2.40)	1.15	(1.15)	2.67	(1.96)	2.24	(1.69)
	Boys	1.70	(1.71)	1.22	(1.12)	2.64	(1.99)	1.91	(1.59)
Total		2.15	(2.03)	1.24	(1.20)	2.88	(2.12)	1.88	(1.57)

Notes. ^a^ Group mean (standard deviation).

**Table A4:** Internal consistencies (Cronbach’s alpha) of SDQ subscales in the 2018 sample.

Sex	Emotional Symptoms	^a^ Conduct Problems	Hyperactivity/Inattention	^b^ Peer Relationship Problems	^c^ Total Difficulties Score
Girls	0.712	0.291	0.642	0.469	0.709
Boys	0.546	0.312	0.627	0.473	0.692
Total	0.656	0.304	0.632	0.470	0.701

Notes. ^a^ Alpha not acceptable, ^b^ not satisfactory. ^c^ Satisfactory alphas here are at least partly due to scale length (20 items), cp. [39].

**Table A5:** Percentages of children and adolescents screened with “borderline/clinical” SDQ results according to gaming group classification in the 2018 sample.

Sex	IGDS Score	^a^ Emotional Symptoms		^b^ Conduct Problems		Hyperactivity/Inattention		^c^ Peer relation-Ship Problems	
		%	**n**	%	**n**	%	**n**	%	**n**
Girls	Normal	14.6	(38)	8.8	(23)	11.9	(31)	13.8	(36)
	At-risk	38.1	(8)	28.6	(6)	23.6	(5)	33.3	(7)
	Pathologic	66.7	(2)	33.3	(1)	33.3	(1)	33.3	(1)
Boys	Normal	10.5	(43)	3.6	(15)	7.5	(31)	15.8	(65)
	At-risk	32.8	(22)	11.9	(8)	28.4	(19)	25.4	(17)
	Pathologic	57.1	(12)	19.0	(4)	33.3	(7)	42.9	(9)
Total	Normal	12.1	(81)	5.7	(38)	9.2	(62)	15.0	(101)
	At-risk	34.1	(30)	15.9	(14)	27.3	(24)	27.3	(24)
	Pathologic	58.3	(14)	20.8	(5)	33.3	(8)	41.7	(10)

Notes. ^a^ Percentage (absolute frequency). Internal consistency ^b^ not acceptable or ^c^ not satisfactory. Age- and sex-sensitive SDQ cut-off scores []; N = 784, n_boys_ = 499, n_girls_ = 285.
